# Plant Growth Promotion Activity of Keratinolytic Fungi Growing on a Recalcitrant Waste Known as “Hair Waste”

**DOI:** 10.1155/2015/952921

**Published:** 2015-11-30

**Authors:** Ivana A. Cavello, Juan M. Crespo, Sabrina S. García, José M. Zapiola, María F. Luna, Sebastián F. Cavalitto

**Affiliations:** ^1^Research and Development Center for Industrial Fermentations (CINDEFI), CONICET La Plata-UNLP, Calle 47 y 115, B1900ASH La Plata, Argentina; ^2^Instituto de Microbiología y Zoología Agrícola, INTA, Nicolás Repetto y De Los Reseros s/n, CP 1686, Hurlingham, Buenos Aires, Argentina; ^3^Comisión de Investigaciones Científicas de la Provincia de Buenos Aires (CIC-PBA), La Plata, Argentina

## Abstract

*Purpureocillium lilacinum* (Thom) Samsom is one of the most studied fungi in the control of plant parasitic nematodes. However, there is not specific information on its ability to inhibit some pathogenic bacteria, fungi, or yeast. This work reports the production of several antifungal hydrolytic enzymes by a strain of* P. lilacinum* when it is grown in a medium containing hair waste. The growth of several plant-pathogenic fungi,* Alternaria alternata*,* Aspergillus niger*, and* Fusarium culmorum*, was considerably affected by the presence of* P. lilacinum*'s supernatant. Besides antifungal activity,* P. lilacinum* demonstrates the capability to produce indoleacetic acid and ammonia during time cultivation on hair waste medium. Plant growth-promoting activity by cell-free supernatant was evidenced through the increase of the percentage of tomato seed germination from 71 to 85% after 48 hours. A 21-day plant growth assay using tomato plants indicates that crude supernatant promotes the growth of the plants similar to a reference fertilizer (*p* > 0.05). These results suggest that both strain and the supernatant may have potential to be considered as a potent biocontrol agent with multiple plant growth-promoting properties. To our knowledge, this is the first report on the antifungal, IAA production and tomato growth enhancing compounds produced by* P. lilacinum* LPSC #876.

## 1. Introduction

Keratin solid wastes are globally recognized as a crucial industrial waste because of the amount produced every year and the environmental problems that they represent due to their limited use and the consequent need of disposal. Argentina's economy is strongly based on agriculture related industries. There are around 50 million livestock and more than 200 tanneries that process about 16 million bovine hides per year. Although hair-saving unhairing processes reduce the organic (COD) load from beamhouse liquid effluent, a new solid residue called “hair waste” is generated, being then necessary for its appropriate disposal. The amount of hair recovered from a bovine hide after this process is about 3% (dry weight based). Therefore, it was estimated that a tannery, processing 25 ton of salted hides per day, produces about 2.5 ton of wet hair (70% moisture) [1, 2]. Nowadays, after the processing of bovine hides, hair wastes are disposed to landfills carrying potent polluting implications.

The main component of hair waste is keratin, a fibrous and insoluble structural protein rich in *β*-helical coils linked through disulfide bridges which render to this protein the resistance to degradation by common proteases like pepsin, papain, and trypsin [3]. Considering its high protein content, this kind of waste could have a great potential. The biotransformation of hair waste could give rise to a partially degraded organic material with high nitrogen content due to extent of the proteolysis when microorganisms act. This new biomaterial could have different potential uses, that is, as a highly digestible protein source for animal feeding, as a raw material in the fertilizing or chemical industry, and as substrate for the production of enzymes such as proteases and keratinases [2, 4, 5].

Although several potential applications are proposed for the utilization of this kind of recalcitrant wastes, the simplest and most appropriate use is as fertilizers, being necessary for the isolation and characterization of keratin-degrading microorganisms not only with keratinolytic activity but also with plant growth-promoting activity [6, 6–9]. From an agrobiochemical point of view, keratinolytic microorganism, which is able to produce antifungal and plant growth-promoting activities, could offer a number of economic and environmental advantages over chemical-based control applications [10].

The aim of this work was to study plant growth-promoting activity of the keratinolytic fungus* Purpureocillium lilacinum* LPSC #876. This fungus is able to produce proteases, keratinases, laminarases, and chitinases enzymes simultaneously under submerged fermentation using hair waste as substrate. It also produces one of the most physiologically active auxins, indoleacetic acid (IAA), and presents several antifungal activities, which turn* P. lilacinum* culture supernatant into a promising candidate for the development of an organic biofertilizer with biological control.

To our knowledge, this is the first report on the antifungal activity, phytohormones production, and plant growth enhancement by* P. lilacinum* LPSC #876.

## 2. Materials and Methods

### 2.1. Chemicals

Laminarin, chitin, N-acetylglucosamine, N-D-galacturonic acid monohydrate (GALA), polygalacturonic acid (PGA) from citrus fruits, and carboxymethylcellulose (CMC) were purchased from Sigma Chemical Co., St. Louis, MO. All other chemicals were commercially available and were of analytical grade.

### 2.2. Microorganism, Inoculum Preparation, and Culture Conditions

*Purpureocillium lilacinum* LPSC #876 (formerly* Paecilomyces lilacinus*), isolated from soils in public places from La Plata, Argentina [11], is a nonpathogenic fungal strain, which is deposited at the Spegazzini Institute fungal culture collection (La Plata National University, Argentina).

*P. lilacinum* was streaked on Potato Dextrose Agar dishes and incubated at 28°C for 10 days. After this period of time, spores were harvested by flooding the agar dish with 10 mL of 0.05% Tween 80 sterile solution and gently stirring the surface with a sterile magnetic bar. Concentration of spore suspension was determined in a Neubauer chamber [12].

Cultures were established in 1,000 mL Erlenmeyer flasks in 200 mL of hair medium containing (per liter) 10 g hair waste, 0.496 g NaH_2_PO_4_, 2.486 g K_2_HPO_4_, 0.0016 g FeCl_3_·6H_2_O, 0.0013 g ZnCl_2_, 0.0010 g MgCl_2_, y 0.0065 mg CaCl_2_, and 7.10 g glucose (pH 5.6) [13]. Hair waste, obtained from a local tannery, was extensively washed with tap water, dried at 60°C for 2 days, and kept at room temperature until used. The culture flasks were autoclaved at 121°C for 15 min for sterilization and then, after cooling, inoculated with 2 × 10^6^ conidia per mL. The cultures were incubated in an orbital shaker at 200 rpm and 28°C. After 6 days of cultivation, the culture was withdrawn and centrifuged (5,000 ×g, 10 min, 4°C), and the supernatant was then used to test some* in vitro* and* in vivo* activities involved in plant growth promotion and biological control.

### 2.3. *In Vitro* Screening of* P. lilacinum* for Its Plant Growth-Promoting and Antifungal Activities

#### 2.3.1. Assay for Indoleacetic Acid (IAA) Production

Production of IAA was investigated in optimal hair waste medium with different concentration of L-tryptophan (0%, 0.02%, and 0.1%). Cultures were performed as was described above and samples were withdrawn every regular period of time and IAA was estimated by mixing 1 mL of Salkowski reagent with 1 mL of culture supernatant followed by measuring absorbance at 530 nm after 30 min of incubation at room temperature. IAA from Sigma was used as standard [14].

#### 2.3.2. Phosphate Solubilization by* P. lilacinum*

Phosphate solubilization was investigated in NBRIP (National Botanical Research Institute's phosphate) growth medium containing 0.5% (w/v) Ca_3_(PO_4_)_2_ as the insoluble phosphate source [10]. Solubilization of phosphate by the microorganism was demonstrated by the production of a halo around the colony.

#### 2.3.3. NH_3_ Production

The production of ammonia due to fungal degradation of hair waste was determined according to the method of Chaney and Marbach using NH_4_(SO_4_)_2_ as standard [15].

#### 2.3.4. Determination of Cell Wall-Degrading Enzymes Activities

*β-1,3*-*Glucanase (laminarase)* activity was measured using laminarin (SIGMA) as substrate. The admixture containing 180 *μ*L of 1% laminarin in 12.5 mM citric acid/6.25 mM Na_2_HPO_4_ buffer (CPB, pH 5.0) and 20 *μ*L of the enzyme preparation was incubated at 37°C for 2 h [16]. After incubation, the released reducing sugars were determined at 660 nm according to Nelson-Somogyi method using glucose as standard [17, 18]. One unit of laminarase activity was defined as the amount of enzyme that catalyzed the release of one micromole of glucose per hour under experimental conditions.

For* chitinase* activity determination, the admixture containing 450 *μ*L of 1% sieved chitin (500 *μ*m > Ø > 100 *μ*m) in CPB (12.5 mM; 6.25 mM, pH 5.0) and 50 *μ*L of culture supernatants was incubated at 37°C for 24 h [19]. The release of reducing sugars was quantified by Nelson-Somogyi method using N-acetyl-D-glucosamine (NAG) as standard. One unit of chitinase activity was defined as the amount of enzyme that releases a reducing power equivalent to one nanomole of NAG per hour under those experimental conditions.

*Polygalacturonase* activity was determined using polygalacturonic acid as substrate. The reaction mixture containing 180 *μ*L of 0.20% polygalacturonic acid dissolved in CPB (12.5 mM; 6.25 mM, pH 5.0) and 20 *μ*L of the culture supernatants was incubated at 37°C for 24 h [20]. The release of reducing sugars was quantified by measuring the rate of increase of galacturonic acid concentrations using Nelson-Somogyi method. One unit of enzyme activity was defined as the amount of enzyme that catalyzed the release of one micromole of galacturonic acid per hour under the given assay conditions.

*Carboxymethyl cellulase* activity was measured using carboxymethyl cellulose as substrate. The reaction mixture containing 180 *μ*L of 1.0% of carboxymethyl cellulose dissolved in CPB (12.5 mM; 6.25 mM, pH 5.0) and 20 *μ*L of the culture supernatants was incubated at 37°C for 24 h [20]. The release of reducing sugars was quantified by measuring the rate of increase of glucose concentrations using Nelson-Somogyi method [17, 18]. One unit of cellulose activity was defined as the amount of enzyme that catalyzed the release of one micromole of glucose per hour under the given assay conditions.

#### 2.3.5. Antifungal Assays

*(1) Antagonistic Properties of P. lilacinum LPSC #876*. A dual culture technique was performed in order to test the ability of* P. lilacinum* to inhibit the growth of several plant-pathogenic fungi (*Alternaria alternata* #25031,* Aspergillus niger* AKU 3302,* Fusarium culmorum* #29,* F. graminearum* FUSKU #117, and* F. graminearum* #206). Plant-pathogenic fungi were grown on potato dextrose agar (PDA, Oxoid) for 10 days at 28°C and then stored at 4°C until used. A 5 mm diameter agar plug containing each fungal mycelium was placed in front of* P. lilacinum* agar plug on PDA surface plates and incubated at 28°C for 5 days. After the plates were incubated, the inhibition of fungal growth was observed around* P. lilacinum* colony. Control plates were prepared by growing each fungus tested in absence of* P. lilacinum*. Dual culture technique for each plant-pathogenic fungus was performed by quintuplicate.

*(2) Antagonist Properties of Cell-Free Supernatant*. Supernatant antifungal activity was estimated via a growth inhibition assay as follows: double concentration PDA was prepared and sterilized and allowed cooling up to 50°C. Two groups of plates were prepared by quintuplicate. In the experimental (*E*) group, equal amounts of concentrated PDA and filtrate sterilized culture supernatant were mixed and poured. In the control (*C*) group, the supernatant was replaced by sterilized distilled water. After plate cooling, a fungal disc (5 mm) of each pathogen was placed on the agar surface. Both groups were incubated at 25°C for 72 h. The diameters of the fungal colonies were recorded twice a day and the averages calculated. For each pathogenic fungus, the colony radial growth rate was estimated from the slope of the linear regression of the colony diameter (cm) on time (h). The percentage of growth inhibition produced by the supernatant was calculated as follows [21]:

(1) $$\begin{array}{c} \text{Inhibition ratio }\left(\;\%\;\right)=\frac{\left(C-E\right)}{C}*100\%, \end{array}$$

where *C* is the average diameter of the colonies of the control groups and *E* is the average diameter of the colonies of the experimental groups.

If the inhibition ratio exceeded 20%, the tested fungus was considered inhibited [22].

### 2.4. *In Vivo* Plant Growth Promotion Assays

#### 2.4.1. Seed Germination Assay

The ability of* P. lilacinum* to promote seedling growth was studied using cell-free supernatant according to Rajkumar and Freitas protocol with slight modifications [23]. Tomato seeds were surface-disinfected with sodium hypochlorite (1.0% v/v) for 10 minutes and washed with sterile distilled water. Equal numbers of seeds (~350) were incubated with 100 mL of sterilized culture supernatant or distilled water, respectively, for 1 h at room temperature. One hundred seeds of each treatment were placed in sterile 0.8% agar plates and incubated at 28°C in the dark. All the treatments were made in triplicate. Visual assessments of seed germination were done daily up to 3 days. Every day, the number of seeds germinated was recorded and the percentage of germination calculated for each treatment. Univariate analysis of variance (ANOVA) was employed on each treatment and tested for its significance using Tukey's Student Range Test (HSD_(0.05)_).

#### 2.4.2. Effect on the Growth of Tomato (*Lycopersicum esculentum* cv. “Superman,” Seminis) Plants

In order to evaluate the biofertilizer capacity of* P. lilacinum* on tomato growth, cell-free culture supernatant was used to irrigate tomato plants. Water and Fähraeus solution (supplemented with nitrogen, 0,5 g/L NO_3 _K) were used as negative and positive control, respectively. Tomato seeds were screened by shape, color, and appearance in order to eliminate bad ones. They were surface-disinfected with 70% ethanol for 5 min and washed (once) with sterile water. Finally, they were immersed in 1.0% sodium hypochlorite for 10 min followed by three washes with sterile water. Seeds were germinated for 4 days at 28°C in the dark on semisolid medium (0.5% agar). Then, germinated seeds were placed in pots with sterile vermiculite as soilless potting media. Four germinated seeds were sown at a suitable depth in all pots, and the pots were placed on a greenhouse at 25°C with a 18/6 h photoperiod. During the experiment, each pot was irrigated with water, Fähraeus solution, or culture supernatant 3 times over a period of 15 days. After day 15, all pots were irrigated with distilled water. After 21 days, the plants were carefully removed from the pots and the root surface was cleaned several times with distilled water. Growth parameters such as fresh and dry weight of roots and shoots were recorded [23]. Univariate analysis of variance (ANOVA) was employed on each treatment and tested for its significance using Tukey's Student Range Test (HSD_(0.05)_).

### 2.5. Experimental Design and Data Analysis

Enzymatic assays and IAA assays were conducted in triplicate and in Completed Randomized Design (CRD). The data were expressed as means ± standard deviations. When it was necessary, results were analyzed by the Design Expert (Stat-Easy, Minneapolis, MN, USA) version 8.0.7.1. trial version software using univariate analysis of variance (ANOVA). Means were compared with Tukey's Student Range Test (HSD_(0.05)_).

## 3. Results and Discussion

The practical employment of keratinase-producing microorganisms is currently being explored within the context of bioconversion for the utilization of keratin wastes at room temperature [24]. These organisms have potential biotechnological applications and could replace the chemical processing used to convert these wastes into useful products.* P. lilacinum* LPS #876 was previously reported as a keratinolytic fungus capable of producing keratinases when hair waste was used as substrate under submerged fermentation. Keratinases of* P. lilacinum* could be used for several purposes as it was stated early [2, 5, 12]. This report describes experiments that suggest that* P. lilacinum* LPS #876 is able to produce a cell-free supernatant that can be considered as a multifunctional crude extract with keratinolytic, antifungal, and plant growth-promoting activities.

### 3.1. *In Vitro* Screening of* P. lilacinum* for Its Plant Growth-Promoting and Antifungal Activities

#### 3.1.1. Indole Acetic Acid Production by* P. lilacinum* in Optimal Hair Medium

**Figure 1:**
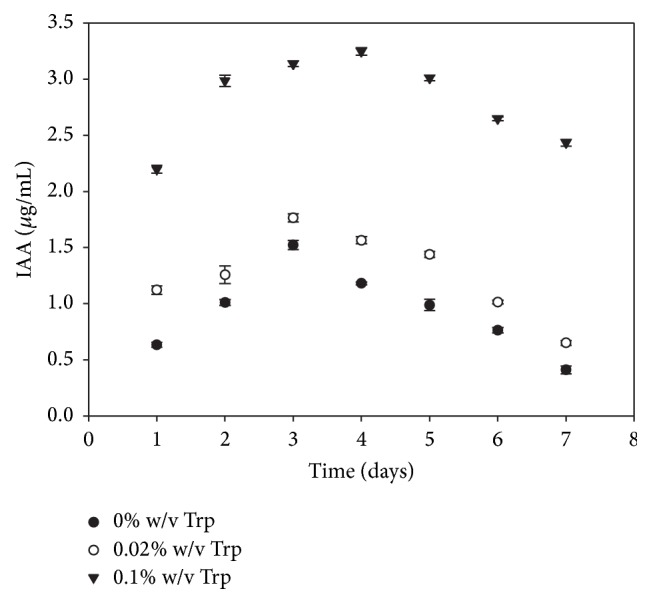
Production of indoleacetic acid by* P. lilacinum* under submerged fermentation containing various concentrations of L-tryptophan. Errors bars (±S.D) are shown when larger than the symbol.

The capability to synthesize IAA is an important feature for a strain to be considered as a plant growth-promoting agent; it is well known that this hormone participates in promotion of plant growth by increasing radical surface of inoculated plants [25]. IAA production was investigated using different concentration of L-tryptophan.  Figure 1 shows that the production of IAA increases as long as the concentration of the precursor increases up to 0.1%. Maximum production of IAA (3.24 ± 0.1 *μ*g/mL) was observed after 4 days of cultivation. Even without L-tryptophan supplementation,* P. lilacinum* was able to produce detectable amounts of IAA, probably due to hair degradation into free soluble amino acids.

Although the concentration of IAA reached by* P. lilacinum* is lesser than the concentration of IAA reached by the keratinolytic bacteria reported by Jeong et al. [8] (114.6 ± 4.8 *μ*g/mL), it is in concordance with those reported for several plant growth-promoting bacteria (PGPB) including the keratinolytic bacteria* Bacillus cereus* TS1 (Table 1). Low levels of IAA can stimulate root elongation, while high levels can stimulate the formation of lateral and adventitious roots [26].

#### 3.1.2. Phosphate Solubilization and Ammonia Production

**Figure 2:**
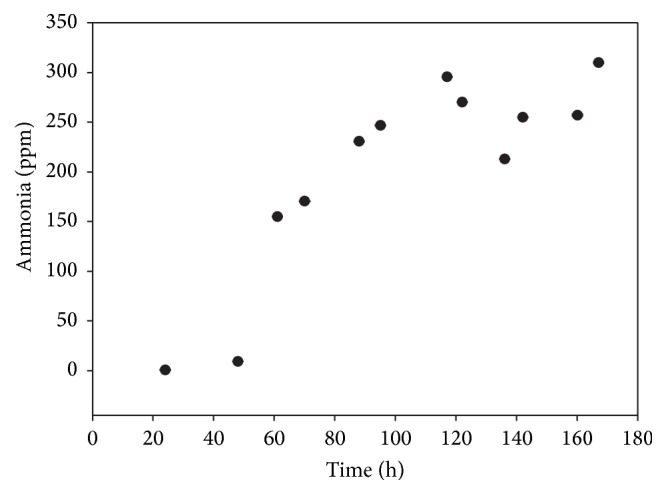
Time course of ammonia production by* P. lilacinum* under submerged fermentation.

*P. lilacinum* could not solubilize insoluble phosphates such as Ca_3_(PO_4_)_2_. As can be seen in Figure 2, during time course of cultivation* P. lilacinum* produces ammonia due to protein degradation, reaching up to 300 ppm after 6 days of cultivation.

#### 3.1.3. Cell Wall-Degrading Enzymes Present in the Culture Supernatant

Among activities tested, culture supernatant of* P. lilacinum* exhibited hydrolyzing activities on laminarin (3.4 U mL^−1^) and chitin (68.1 U mL^−1^); however, nonenzymatic activities against PGA and CMC were detected (Table 2).

*Purpureocillium lilacinum* species are known to be good sources of degradative enzymes such as proteases, keratinases, and chitinases, being the first report of laminarases production by this strain [27–29]. These enzymes provide a remarkable metabolic versatility, enabling the fungus to grow on a wide variety of substrates.

Recent reports have demonstrated that exposure of certain fungal hydrolyzing enzymes such as chitinases, proteases, cellulases, and glucanases to certain fungi resulted in the degradation of most fungal cell walls, the production of these enzymes being an important mechanism of fungal growth inhibition [16, 20, 30, 31]. Jeong et al. [8] have extensively worked with keratinolytic bacteria and their plant growth promotion activities. Among cell wall-degrading enzymes studied, they report the production of amylases, cellulases, pectinases, lipases, and proteases on Petri dish-based assays by keratinolytic bacteria* Bacillus subtilis* S8,* Xanthomonas* sp. P5, and* Stenotrophomonas maltophilia* R13.

#### 3.1.4. Antagonistic Properties of* P. lilacinum* LPSC #876

**Figure 3:**
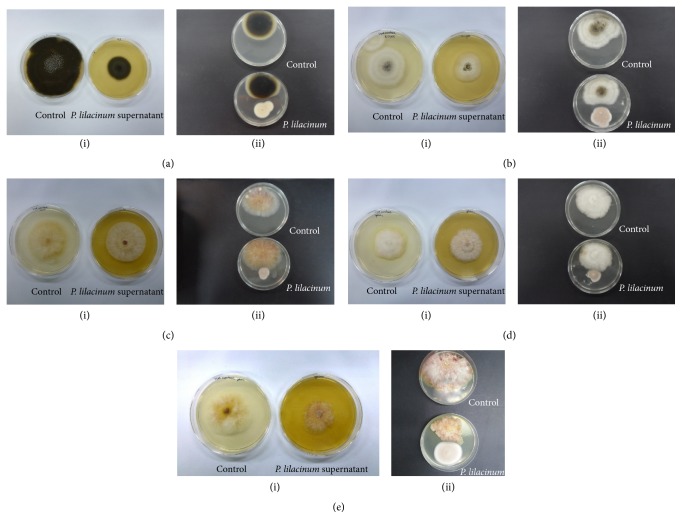
Antifungal activity against fungal phytopathogens: (a)* Alternaria alternata*, (b)* Aspergillus niger*, (c)* Fusarium culmorum*, (d)* Fusarium graminearum* FUSKU #177, and (e)* F. graminearum* #206. (i) Supernatant antifungal activity and (ii) dual culture technique.

*Purpureocillium lilacinum* (Thom) Samsom is one of the most studied fungi, used in the control of plant parasitic nematodes. Under greenhouse and field conditions, it has been found infecting 50–100% of plant parasitic nematodes such as* Meloidogyne incognita* [32, 33],* M. javanica* [34], and* Heterodera avenae* Wollenweber [35],* Radopholus similis* [36], and* Rotylenchulus reniformis* [37]. However, there is not specific information on its ability to inhibit some pathogenic bacteria, fungi, or yeast. In the present study,* P. lilacinum* LPSC #876 was screened for antifungal activity* in vitro* against some phytopathogenic fungi using dual culture assay on PDA plates. As can be seen in Figures 3(a)–3(e),  * P. lilacinum* showed a wide range of antifungal activity against* A. alternata*,* A. niger*,* F. culmorum*, and* F. graminearum #206* but could not control the growth of* F. graminearum* FUSKU #117.

According to Porter,* P. lilacinum* exhibits inhibition type C. In this type of inhibition, the space between the two colonies, while very narrow, is clearly marked [38].

#### 3.1.5. Antagonism Properties of Culture Supernatant

**Figure 4:**
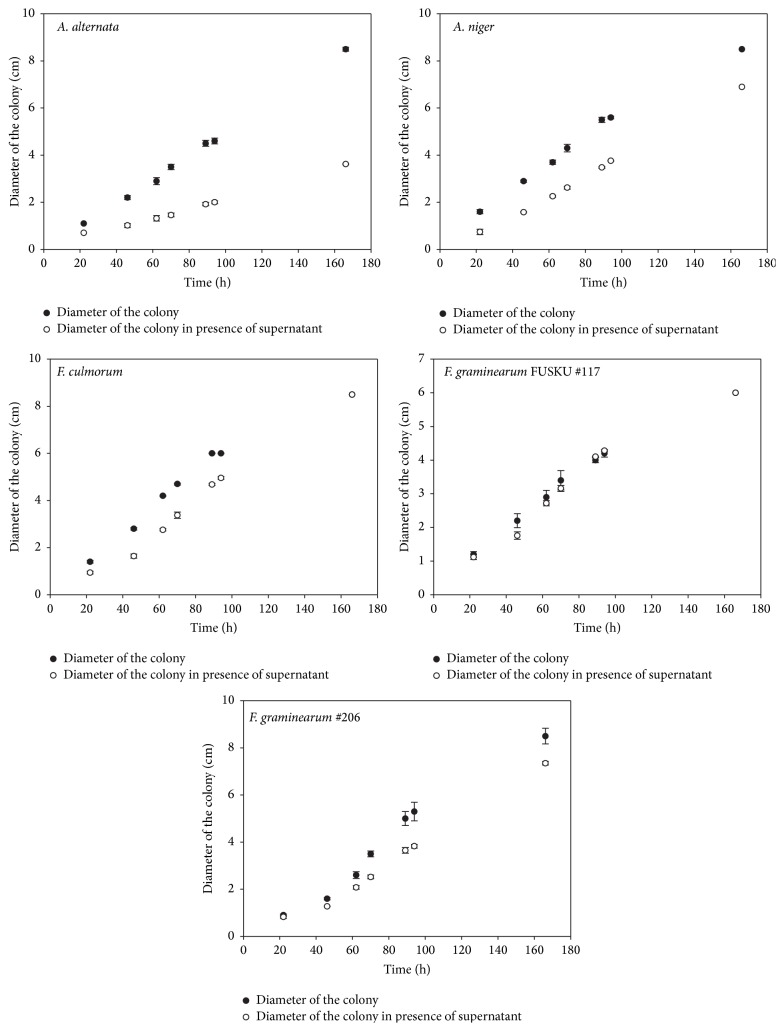
Growth rate of* A. alternata*,* A. niger*,* F. culmorum, F. graminearum* FUSKU #117, and* F. graminearum* #206 in absence (∙) and presence of the supernatant (∘) represented as diameter of the colony (cm) versus time (h). Results represent the means of five experiments, and bars indicate ±standard deviation.

Culture supernatant antifungal activity against selected soil-borne phytopathogens was estimated via a growth inhibition assay. In Table 3 and Figures 3 and 4, the growth rate of each fungus in absence and presence of culture supernatant as well as the inhibition ratio (%) is shown. Except for* F. graminearum* FUSKU #117, all other pathogenic strains were controlled by cell-free supernatant with inhibition ratios higher than 25%.

The antagonistic phenomena against fungi can be explained by several mechanisms, including antibiosis and parasitism. In some cases, hydrolytic enzymes such as chitinases, glucanases, or proteases may act against the fungal cell wall [39]. Stefanova et al. [40] used filtrates from liquid cultures of several isolates of* Trichoderma* sp. Some of these isolates produce nonvolatiles metabolites with antifungal activity which reduce* Phytophthora nicotianae* and* Rhizoctonia solani* growth. Gousterova et al. [41] obtained a protein-rich hydrolysate from feather waste using a mixed culture of thermophilic actinomycete strains which was tested for antifungal activity against some common plant-pathogenic fungi (*Fusarium solani*,* F. oxysporum*,* Mucor* sp., and* Aspergillus niger*). Meanwhile, Mishra et al. [42] reported the effect of cell-free culture filtrates of* T. viride* on different common fungal pathogens, controlling the growth of* R. solani*,* S. rolfsii*,* Macrophomina phaseolina*, and* Colletotrichum capsici* but neither* A. alternata* nor* Pythium aphanidermatum*.

The antifungal activity of* P. lilacinum*'s culture supernatant against phytopathogenic fungi could be attributed to the presence of several hydrolytic enzymes as well as ammonia in the supernatant. The role of ammonia in biocontrol has been described by Pavlica et al. [43]: ammonia is the only gas present in sufficient concentrations in soil to inhibit soil fungi. Although Pavlica et al. [43], Kim et al. [44], and Mishra et al. [42] worked with culture filtrates, none of them measured cell wall-degrading enzymes that could be present in those filtrates. Chang et al. [22] reported that culture supernatant of* B. cereus* QQ308 grown on shellfish chitin wastes exhibited a similar enzymatic activities profile with hydrolyzing activity against chitin, casein, and glycol chitosan and is capable of inhibiting the growth of several soil-borne fungal plant pathogens including* F. oxysporum*,* R. solani*, and* P. ultimum*.

### 3.2. *In Vivo* Plant Growth-Promoting Activity of Cell-Free Supernatant

#### 3.2.1. Effect of Culture Supernatant on Tomato Seed Germination and Tomato Plant Growth

The effect of* P. lilacinum*'s supernatant on tomato seed germination is shown in Table 4. After 48 h of incubation, seed germination increased from 71.3 ± 4.7 to 84.7 ± 2.1% when the seeds were treated with the supernatant. Similar increase was observed by Sivakumar et al. [45] using feather hydrolysates (from 42.1 to 54.8%).

**Figure 5:**
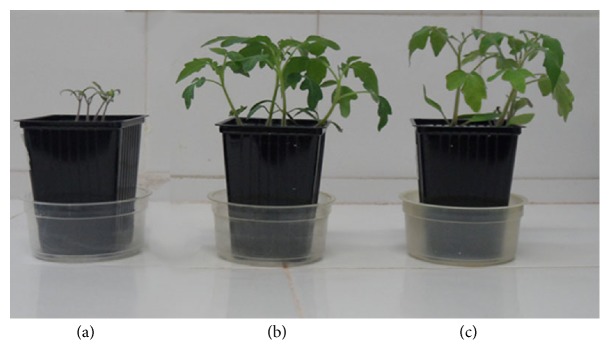
Tomato plants after 15 days of first irrigation. From right to left: (a) water, (b)* P. lilacinum* hydrolysate, and (c) Fähraeus solution.

Tomato plant growth promotion was studied using a 21-day plant growth assay. Water and Fähraeus solution (supplemented with nitrogen) were used as control. In terms of fresh root and shoot weights, it can be seen that cell-free supernatant showed similar effect as that of the reference fertilizer on the growth of tomato plants (Figure 5 and Table 5) (*p* > 0.05).

Similar results were reported by Kim et al. [44] and Esfahani and Pour [34]. Both authors report an improvement in growth of plants when they compared their keratin hydrolysates with reference fertilizers.

## 4. Conclusion

Current trends in agriculture are focused on the reduction of the use of synthetic pesticides and inorganic fertilizers, forcing the search of alternative ways to improve a more sustainable agriculture. It was previously reported that* P. lilacinum* can grow and degrade a recalcitrant waste producing a crude extract with proteolytic activity. In this work, features dealing with plant growth-promoting activity were investigated. Functional traits such as IAA and hydrolytic enzymes production, a broad-spectrum antifungal activity and ammonification, may give to this microorganism and its cell-free supernatant a potential application as biostimulant and biocontrol agent as well as biofertilizer. To our knowledge these characteristics of* P. lilacinum* have not been described previously.

## Figures and Tables

**Table 1:** IAA production of several reported plant growth-promoting microorganisms.

Microorganism	Tryptophan concentration (% w/v)	IAA (*μ*g/mL)	Reference
*P. lilacinum* LPSC #876	0.1	3.24	
*Gluconacetobacter diazotrophicus* UAP 5701	0.01	1.07	Fuentes-Ramirez et al. (1993) [46]
*G. diazotrophicus *UAP 7308	0.01	1.12	Fuentes-Ramirez et al. (1993) [46]
*Azospirillum brasilense* CBG	0.1	0.75	Hernández-Mendoza et al. (2008) [47]
*G. diazotrophicus* 4-02	0.01	1.8	Rodríguez Cheang et al. (2005) [48]
*G. diazotrophicus* PAl-5	0.01	2.2	Rodríguez Cheang et al. (2005) [48]
*Pseudomonas fluorescens* AK-1	0.01	4.0	Karnwal (2009) [49]
*Bacillus cereus TS1*	0.04	2.12	Sivakumar et al. (2012) [45]
*A. diazotrophicus* L1	0.02	1.51	Patil et al. (2011) [50]

**Table 2:** Enzymes activities shown by culture supernatant of *P. lilacinum* LPSC #876 using various substrates.

Enzymes	Substrates	Concentration (w/v)	Enzyme activity (UmL^−1^)
*Laminarase*	Laminarin	1.0%	3.4 ± 0.2
*Chitinase*	Chitin	1.0%	68.1 ± 0.3
*Polygalacturonase*	Polygalacturonic acid	0.2%	nd
*Cellulase*	CM-cellulose	1.0%	nd

n.d.: not detectable.

**Table 3:** Antagonistic effect of *P. lilacinum *supernatant on growth rate and percentage of mycelial growth inhibition of several phytopathogens.

Phytopathogen	Growth rate (cmday^−1^)	Growth rate in presence of *P. lilacinum* supernatant (cmday^−1^)	Inhibition rate (%)
*A. alternata* #25031	1.20 ± 0.08	0.50 ± 0.01	57.0
*A. niger*	1.16 ± 0.02	1.03 ± 0.01	27.0
*F. culmorum* #29	1.80 ± 0.05	1.30 ± 0.01	34.0
*F. graminearum* FUSKU #117	0.80 ± 0.04	0.80 ± 0.04	—
*F. graminearum* #206	1.30 ± 0.07	1.10 ± 0.02	27.0

**Table 4:** Effect of culture supernatant on seed germination. Results are expressed as mean ± SD; *n* = 300 (*n*: number of seeds tested for each treatment).

	Control (%)	Treatment with hydrolysate (%)
48 h of incubation	71.3 ± 4.7	84.7 ± 3.5
72 h of incubation	79.0 ± 1,0	86.7 ± 2.1

**Table 5:** Effect of culture supernatant of *P. lilacinum* LPSC #876 on tomato seedling growth.

Growth parameters	Water	Hair hydrolysate	Fähraeus solution
Above-ground vegetation dry weight (g)	0.0218 ± 0.002	0.3927 ± 0.053	0.4182 ± 0.036
Root dry weight (g)	0.0282 ± 0.002	0.2093 ± 0.036	0.1879 ± 0.022
